# Accuracy of Maternal Reports of Young Children’s Dental Disease Status: Avon Longitudinal Study of Parents and Children †

**DOI:** 10.3390/dj8010008

**Published:** 2020-01-10

**Authors:** Aderonke A. Akinkugbe, Tegwyn H. Brickhouse, Dipankar Bandyopadhyay, Marcelle M. Nascimento

**Affiliations:** 1 Department of Dental Public Health and Policy, School of Dentistry, Virginia Commonwealth University, Richmond, VA 23298, USA; thbrickhouse@vcu.edu; 2 Institute for Inclusion, Inquiry, and Innovation, Virginia Commonwealth University, Richmond, VA 23298, USA; 3 Department of Biostatistics, School of Medicine, Virginia Commonwealth University, Richmond, VA 23298, USA; bandyop@vcuhealth.org; 4 Department of Restorative Dental Sciences, University of Florida College of Dentistry, Gainesville, FL 32610, USA; mnascimento@dental.ufl.edu

**Keywords:** self-reports, public health dentistry, dental disease, dental care, ALSPAC

## Abstract

**Background:** Parental reports of their children’s health status is integral to pediatric medical and dental care. Therefore, understanding the accuracy of such reports is vital. Our objectives were to (1) assess the correlation between maternal reports of their children’s indicators of caries experience (subjective assessment) and actual caries status determined by oral examination (objective assessment), and (2) identify potential modifiers of this correlation. **Methods:** Longitudinal data from the Avon longitudinal study of parents and children (*n* = 1429) was used to assess the correlation between maternal reports of the number of missing and filled teeth of children aged 38 months, 54 months, and 5.5 years and clinical oral examinations of decayed, missing and filled teeth conducted when the same children were 31, 43 months, and five years of age. Homogeneity chi-square tests assessed differences in correlations according to sociodemographic factors. **Results:** Overall, we found a statistically significant correlation that was weak to moderate in magnitude. Maternal reports of missing teeth at 38 months was significantly correlated with decayed teeth, 0.27 (*p* < 0.001); missing teeth, 0.23 (*p* < 0.001), and the decayed, missing and filled (dmft) index, 0.35 (*p* < 0.001) based on oral examination at 31 months. A maternal report of filled teeth at 54 months was significantly correlated with decayed teeth, 0.30 (*p* < 0.001); filled teeth 0.30 (*p* < 0.001), and dmft 0.40 (*p* < 0.001) at 43 months. Mothers tended to underestimate the extent of missing and filled teeth in their children irrespective of the child’s age, but the extent of underestimation was greater among younger children. Maternal age, education level, and whether the child had ever visited a dentist were significant modifiers of subjective and objective caries assessments. **Conclusions:** From a clinical and dental public health perspective, our findings of a weak to moderate correlation of maternal assessments of their children’s caries experience may be concerning when reporting the burden of dental diseases in large population studies or for surveillance purposes that rely on self-reported measures and must therefore be utilized with caution because of the potential to result in underestimated disease burden.

## 1. Introduction

Dental caries is a common chronic disease estimated to affect a quarter of U.S. children ages 2–5 years [1]. Five-times more common than asthma, dental caries is responsible for many emergency room visits, pain, suffering, missed school days [2], and poor oral health related quality of life [3] in the affected child with negative impacts on the quality of life of families [4].

Several measurement criteria are available for assessing dental caries [5]: the most common is the objective assessment of cavitated carious lesions using either the number of decayed, missing, and filled teeth (DMFT) or surfaces (DMFS) [6,7] and the International Caries Detection and Assessment System (ICDAS) [8]. The ICDAS records caries on a continuum comprising healthy tooth surface, incipient and cavitated lesions. The ICDAS system uses two decisions to classify each tooth surface; the first decision uses nine criteria to determine if a tooth surface is sound, sealed, restored, crowned, or missing while the second decision classifies the caries status of a tooth surface on an ordinal scale [8]. While the ICDAS method is preferred for caries assessment because of the clearly defined criteria for visual detection of caries, it might be a daunting task in large scale epidemiologic studies when considering the 182 possible tooth surfaces to be evaluated and the often-limited resources of such studies. 

An alternative to objective caries assessments by trained examiners is parental reports. Parental report is integral to pediatric medical and dental care and studies in medicine indicate that parental reports of childhood asthma were comparable to medical records of an asthma diagnosis [9] and that parents were able to correctly report prescribed asthma [10,11] and attention deficit hyperactivity disorder [12] medications of their children. Moreover, mothers accurately reported their children’s immunization status when compared to immunization records [13] and retrospectively reported child birth weight, gestational age, and preterm birth delivery status when compared to medical birth records [14]. In dentistry, however, the accuracy of parental reports is less certain. Studies have reported a positive correlation between mothers’ perception of the dental care need of their children and the actual oral health status determined by clinical oral examination [15,16]. Other studies have found maternal awareness of unrestored tooth decay in their children to underestimate actual unrestored tooth decay [17], while another found maternal reports to underestimate the amount of dentifrice used by children while tooth brushing [18,19]. 

While subjective measures (including self-report) may be of value, they are notorious for being of higher sensitivity than specificity, a desirable quality when the goal is to estimate the prevalence of a given condition. In etiologic research, however, a high specificity is preferred to sensitivity [20]. Nonetheless, in studies that have validated self-reported measures, association measures of etiologic research were found to be valid irrespective of the sensitivity of the self-reported measure. Previous oral health studies of parental reports have investigated caregivers’ assessments of the oral health status (excellent, very good, good, fair, or poor) of their children against clinically determined oral health treatment needs [15,16]. In contrast, this study seeks to (1) assess the correlation between mothers’ report of their children’s indicators of caries experience and actual caries status and (2) assess whether certain sociodemographic factors are modifiers of subjective (maternal assessment) and objective (DMFT determined by oral examination) reports. More specifically, the aims of this study were to quantify the correlation between maternal reports of offspring missing teeth, and dental restorations with the DMFT index at different time points, and to assess if these correlations differ by certain sociodemographic characteristics.

## 2. Methods

### 2.1. Data Source and Population

The Avon Longitudinal Study of Parents and Children (ALSPAC) is a population based prospective cohort study of environmental and genetic factors on the health and development of children. Pregnant residents of Avon, UK with an expected date of delivery between 1 April 1991 and 31 December 1992 were eligible to participate [21,22]. Of those recruited at baseline, 63% completed study questionnaires. Response rates were 89%, 75%, and 69% at 2, 3, and 5 years, respectively [23]. A random 10% sample of children born in the last 6 months (June–December 1992) of the study were invited to participate in a sub-study called the “children in focus” (CIF). The CIF sub-study allowed for an extensive examination (including oral health assessments) of children in a way that could not be done using questionnaires completed by the parents [21,24]. Informed consent for the use of the data collected via questionnaires and clinics was obtained from participants following the recommendations of the ALSPAC Ethics and Law Committee at the time of data collection. The current study was approved by the Institutional Review Board of Virginia Commonwealth University as exempt (# HM20011742) on 26 October 2017. 

### 2.2. Assessment of Dental Caries

#### 2.2.1. Maternal Reports

As part of ALSPAC, mothers reported factors related to their offspring on questionnaires they received by mail when their children were the following ages: 4 weeks, 6, 15, 24, 38 months, 54 months, and 5 years and 5 months, 6 years and 5 months, and 8 years and 7 months. Oral health specific reports included the number of teeth, dental visits, frequency of tooth brushing as well as missing teeth and dental restorations (fillings). Maternal reports of their children’s oral health status at ages 38 months, 54 months, and 5.5 years were used in the present study so as to closely align with the ages when the oral examinations were conducted. 

#### 2.2.2. Oral Examinations

Oral health assessments were conducted at 31, 43, and 61 months of age in the CIF focus sample. Oral examinations, which included a visual examination to detect cavitated carious lesions and existing restorations, were performed by dentists at ages 31 months, 43 months, and by trained health examiners when the CIF sample were 5 years old. Training of the health examiners was done in six tutorial sessions totaling 16 hours, accompanied by an hour-long session of mock examination and replication on 30 children. Reported kappa statistics for the examiners was 0.63 [24]. The number of decayed teeth, missing teeth, filled teeth separately and the number of decayed, missing, and filled teeth (DMFT) index, i.e., caries experience was recorded, and data are available for 99.1% of the CIF sample. 

### 2.3. Assessment of Covariates

Maternal factors: were self-reported or abstracted from mothers’ medical records [22]. Those considered for this study were: (a) age at delivery, categorized into 15–24; 25–34; 36–44 years, (b) education level, categorized as O-level or less; A-level; college degree, and (c) race (white; non-white).

Child-specific factors: were obtained from maternal reports on mailed-in questionnaires or determined during CIF clinical evaluations [21,23]. Child-level covariates considered for this study include (a) sex (male, female), (b) age at oral examination, (c) race (white; non-white), and (d) whether the child had ever visited a dentist (yes or no) and birth order (first vs. second or higher).

The study website contains detail of all the data that is available through a fully searchable data dictionary and variable search tool at: http://www.bristol.ac.uk/alspac/researchers/our-data/.

### 2.4. Statistical Analysis

Data analysis was restricted to the CIF sample and started with an examination of the overall distribution of the maternal and child related characteristics. Frequencies and relative frequencies were reported for categorical variables and mean and interquartile range (IQR) for continuous variables with differences assessed with chi-square tests and *t*-tests, respectively.

Maternal reports of child decay were not asked on the mailed-in questionnaire; however, maternal reports of missing teeth and filled teeth were collected on the child-specific questionnaires mothers filled out when the children were 38 months, 54 months, and 5.5 years old. Even though maternal reports of decayed teeth were not collected, teeth may have been missing or filled due to caries, thus we estimated the correlation between maternal reports of missing teeth and filled teeth separately with objectively determined decayed teeth, missing teeth, filled teeth, and the dmft index. These correlations were further assessed for the different time periods for which data were available, 38 months, 54 months, and 5.5 years for maternal reports against examiner reports at 31 months, 43 months, and 5 years, to allow for a more robust assessment. Because sample sizes for maternal reports and objective assessments were often not the same and to maximize the total sample, correlation coefficients were preferred to kappa statistics. Nevertheless, when possible, kappa statistics [25] were estimated.

Even though examiner reports of decayed teeth, missing teeth, filled teeth, and the dmft index were reported as counts, we calculated a binary variable to indicate whether a child had decayed teeth, missing teeth, filled teeth, or a dmft index of 1 or more (i.e., person-level estimates) to allow for easy comparison and the estimation of correlation coefficients with maternal reports that were collected on the person-level. We interpreted correlation coefficient of 0.10 to 0.30 as weak correlation, 0.30 to 0.50 as moderate and greater than 0.50 as strong correlation [26]. Given that these thresholds refer to linear associations, caution must be exercised with their use in this context. Nevertheless, it is generally accepted that higher correlation coefficients with small *p*-values indicate departure from a null hypothesis of no correlation. In other words, small (i.e., significant) *p*-values suggest strong correlation between measures. 

To assess if maternal or child specific characteristics affected the level of correlation between subjective and objective assessments at the different time points, we explored four variables (maternal age at delivery, educational attainment, child sex, and dental visit), among variables previously investigated [15,16] as potential effect modifiers, setting the threshold for statistical heterogeneity at *p* ≤ 0.05. A significant departure from homogeneity (i.e., *p* ≤ 0.05) suggests that the correlations under study is not uniform across the subgroups being investigated [27]. Data analysis was conducted in SAS vs. 9.4 (SAS Institute, Cary, NC, USA). 

## 3. Results

The mean (IQR) of age at delivery was 29 (26–32) years and 75% of the mothers were aged between 25–35 at delivery. Most had less than a college degree (85%) and the majority (98%) were white. Fifty-four percent of the CIF were male and 82% had ever visited a dentist (Table 1).

Of 1211 children at 38 months, <5 and 5 (0.41%) had missing and filled teeth, respectively, based on maternal reports. Correspondingly, of 1154 children at 54 months of age, 38 (3.29%) and 60 (5.20%) had missing and filled teeth, respectively, while 37 (3.45%) and 102 (9.51%) had missing and filled teeth respectively among 1073 children at 5.5 years (Table 2). A total of 1029 children had complete oral examination reports at 31 months, of whom 21 (2.04%) had decayed teeth, 7 (0.68%) had missing teeth, and none had filled teeth with a corresponding number of children with dmft index ≥1 of 28 (2.72%) (Table 2). Of 1026 children with dental examinations at 43 months, 126 (12.3%), 17 (1.66%), and 15 (1.46%) had decayed, missing, and filled teeth, respectively, with 146 (14.2%) having a dmft index of ≥1 (Table 3). Among the 982 children with oral examination data at 5 years, 240 (24.4%) had decayed teeth, 54 (5.90%) had missing teeth, and 64 (6.52%) had filled teeth with a corresponding total number of children with dmft index ≥1 of 233 (25.5%) (Table 4). The total number of children with decayed, missing and filled teeth does not correspond to the total with dmft index ≥1 for the 43 month and 5-year time period because a child who had both decayed and filled teeth is counted twice, once under the decayed teeth group and once under the filled teeth group but only counted once under the dmft index.

### 3.1. Pearson Correlations

Maternal reports of missing teeth at 38 months were significantly correlated with decayed teeth, 0.27 (*p* < 0.001); missing teeth, 0.23 (*p* < 0.001) and the dmft index, 0.35 (*p* < 0.001) based on oral examination at 31 months while no significant correlation was observed between maternal reports of filled teeth and any of the examiner determined measures during the same time period (Table 2). Maternal reports of missing teeth at 54 months were positively and significantly correlated with decayed teeth, 0.26 (*p* < 0.001); missing teeth, 0.30 (*p* < 0.001) and dmft index, 0.30 (*p* < 0.001). Similarly, maternal reports of filled teeth at 54 months were significantly correlated with decayed teeth, 0.30 (*p* < 0.001); filled teeth 0.30 (*p* < 0.001) and dmft 0.40 (*p* < 0.001) at 43 months (Table 3). Maternal reports of missing teeth at 5.5 years were significantly correlated with examiner determined missing teeth at 5 years, 0.49 (*p* < 0.001) and the dmft index, 0.30 (*p* < 0.001), while maternal reports of filled teeth at 5.5 years were significantly correlated with examiner determined decayed teeth, 0.4 (*p* < 0.001); filled teeth, 0.46 (*p* < 0.001) as well as the dmft index, 0.46 (*p* < 0.001) based on oral examination at 5 years (Table 4).

### 3.2. Kappa Statistics

The estimated kappa for maternal report of missing teeth at 38 months and objectively assessed missing teeth at 31 months was 0.22. Kappa statistics for missing teeth increased to 0.29 at the 54-month time point and to 0.46 at the 5.5-year time period. Kappa statistics for filled teeth were 0.27 and 0.45 at 54 months and the 5.5-year time periods respectively (results not shown).

### 3.3. Effect Modifiers

Several of the variables assessed were identified as potential modifiers of the correlations between subjective and objective reports. For instance, maternal education beyond O-level was significantly correlated with accurate maternal assessments of missing and filled teeth in their children during the first two time periods (38 months and 54 months). However, this significant modification of subjective and objective reports by maternal education was rendered non-significant at the third time period when the children were five years old. Similarly, mothers of children who had never seen a dentist had significantly higher correlation for reporting missing teeth at 54 months and 5 years, but no difference in reporting was observed for filled teeth during these same time periods (Table 5).

## 4. Discussion

This study aimed at evaluating the concordance between maternal reports of caries experience in the child, and actual dental disease status determined by clinical oral examination was consistent with statistically significant correlation coefficients and kappa statistics that were weak to moderate in magnitude. Mothers tended to underestimate dental disease in their children irrespective of the child’s age, a finding that is consistent with a previous report of underestimated dental disease status by caregivers when compared to actual dental disease status [17]. Furthermore, our findings indicate that, as the children got older, maternal reports of their child’s dental disease status improved i.e., higher and statistically significant correlation with objective assessment, which is also in line with a previous study that found caregivers’ assessments of their children’s oral health status to correlate poorly with examiner determined clinical treatment needs for younger children but improved among older children [15], but contrary to another study in a high dental caries risk population that found higher concordance among younger children [16]. Nevertheless, our overall finding of a statistically significant correlation between objective and subjective caries experience status is similar to the general findings of positive statistical correlation of objective and subjective oral health status previously reported [15,16,28,29]. 

The magnitude of the correlation in this study was modest, a surprising finding given that mothers had to recall their children’s missing and filled teeth status at a time period after clinical oral examination had occurred. It therefore is possible that oral examiners did not inform the mothers of their children’s dental disease status or perhaps mothers were unable to recall children’s caries status for other reasons. This further suggests that caregivers may not have the necessary expertise to readily identify signs of dental disease in their children. While we were unable to directly assess decayed teeth, the study by Goodman et al. [17] indicated that caregivers consistently underestimated unrestored tooth decay in their children, similar to what we found with missing teeth and filled teeth which were consistently underreported by mothers.

Previous studies have reported differences in concordance between subjective and objective measures based on income [17], child’s age [15,16], and dental insurance status [16]. This study likewise found significant differences in estimated correlation between subjective and objective reports according to maternal education (a proxy measure for income); child sex, maternal age; and whether the child had ever visited a dentist. From a clinical and dental public health research perspective as well as from a public health planning for health care funding of programs for children, our findings of a weak to moderate correlation of maternal assessments of their children’s dental disease status may be concerning when reporting the burden of dental disease in large population studies that rely on parental reports but may not be a source of significant concern in etiologic research.

## 5. Limitations and Strengths

Maternal reports of decayed teeth were not collected thus, we were unable to directly evaluate the correlation between this measure against examiner determined decayed teeth. This study did not utilize the full spectrum (i.e., counts) of decayed, missing, and filled teeth but rather dichotomized these measures to allow for easy comparison with maternal reports. Furthermore, the missing teeth component may be misleading given that we were unable to differentiate missing due to caries from missing resulting from exfoliation of primary teeth that is not disease related. The majority of the samples were white and first-born children, thus results may not generalize to minority populations and higher order births. A major strength of this study is that it explores an important question of the accuracy of caregivers’ report of dental disease in their child. This is an important area of inquiry because the vast majority of dental public health, surveillance, and research is based on self-reported data. The relatively large sample size and the availability of data on this unique cohort of the same mother-child pairs at multiple time points allowed us to assess how self-reports may have improved over time when compared to objective reports of dental disease in the child.

## 6. Conclusions

Mothers underestimated the caries experience status irrespective of their children’s age, but these underestimates improved over time among older children. In other words, caregivers tended to report less dental disease in younger as opposed to older children. Therefore, maternal reports of children’s dental disease for public health and surveillance purposes must be utilized with caution because of the potential to result in underestimated disease burden.

## Figures and Tables

**Table 1:** Demographic characteristics of mothers and their children at baseline: Avon Longitudinal Study of Parents and Children.

	Number (N)	Percent (%)	*p*-Value
**Maternal Characteristics**			
**Age at Delivery, Mean (IQR)**	29	26–32	<0.0001
**Age at Delivery (years)**			<0.0001
15–24	240	17	
25–35	1072	75	
36–44	116	8	
Missing	1		
**Education**			<0.0001
O-level or less	819	60	
A-level	344	25	
College degree	199	15	
Missing	67		
**Race**			<0.0001
White	1333	98	
Non-white	31	2	
Missing	65		
**Children Characteristics**			
**Sex**			0.002
Male	772	54	
Female	657	46	
**Age, Months, Mean (IQR)**	37	38–38	<0.0001
**Race**			<0.0001
White	1293	96	
Non-white	47	4	
Missing	89		
**Ever Visited a Dentist**			<0.0001
Yes	990	82	
No	221	18	
Missing	218		
**Birth Order**			<0.0001
First	1411	99	
Second or higher	18	1	

**Table 2:** Measures of correlation between caregivers’ assessments of children’s dental disease status at 38 months, 54 months and 5.5 years and clinically determined oral disease status at 31 months of age.

		Examiner Determined at 31 Months Clinic Visit (*n* = 1029)			
		Decayed Teeth	Missing Teeth	Filled Teeth	Dmft ≥1
**Caregiver Report**	N (%)	21 (2.04)	7 (0.68)	0 (0)	28 (2.72)
**38 months**	1211	Pearson correlation (*p*-value)			
Missing teeth	<5 *	0.267 (<0.001)	0.232 (<0.001)	-	0.350 (<0.001)
Filled teeth	5 (0.41)	−0.009 (0.8)	−0.005 (0.9)	-	−0.011 (0.7)
**54 months**	1154				
Missing teeth	38 (3.29)	0.249 (<0.001)	−0.015 (0.7)	-	0.209 (<0.001)
Filled teeth	60 (5.20)	0.139 (<0.001)	−0.020 (0.6)	-	0.110 (0.001)
**5.5 years**	1073				
Missing teeth	37 (3.45)	0.184 (<0.001)	−0.016 (0.6)	-	0.143 (<0.001)
Filled teeth	102 (9.51)	0.050 (0.14)	0.061 (0.07)	-	0.077 (0.03)

* corresponding percentages not reported because of the small cell size. Dmft-decayed missing and filled teeth index.

**Table 3:** Measures of correlation between caregivers’ assessments of children’s dental disease status at 38 months, 54 months, and 5.5 years and clinically determined oral disease status at 43 months of age.

		Examiner Determined at 43 Months Clinic (*n* = 1026)			
		Decayed Teeth	Missing Teeth	Filled Teeth	Dmft ≥1
**Caregiver Report**	N (%)	126 (12.3)	17 (1.66)	15 (1.46)	146 (14.2)
**38 months**	1211	Pearson correlation (*p*-value)			
Missing teeth	<5 *	0.094 (0.003)	0.444 (<0.001)	−0.007 (0.8)	0.140 (<0.001)
Filled teeth	5 (0.41)	−0.017 (0.6)	−0.006 (0.8)	0.185 (<0.001)	0.048 (0.14)
**54 months**	1154				
Missing teeth	38 (3.29)	0.264 (<0.001)	0.295 (<0.001)	0.034 (0.3)	0.259 (<0.001)
Filled teeth	60 (5.20)	0.300 (<0.001)	0.047 (0.2)	0.330 (<0.001)	0.354 (<0.001)
**5.5 years**	1073				
Missing teeth	37 (3.45)	0.211 (<0.001)	0.072 (0.03)	0.029 (0.4)	0.187 (<0.001)
Filled teeth	102 (9.51)	0.295 (<0.001)	0.075 (0.03)	0.086 (0.01)	0.314 (<0.001)

* corresponding percentages not reported because of the small cell size. Dmft-decayed missing and filled teeth index.

**Table 4:** Measures of correlation between caregivers’ assessments of children’s dental disease status at 38 months, 54 months, and 5.5 years and clinically determined oral disease status at 5 years of age.

		Examiner Determined at the 5-Year Clinic (*n* = 982)			
		Decayed Teeth	Missing Teeth	Filled Teeth	Dmft ≥1
**Caregiver Report**	N (%)	240 (24.4)	54 (5.90)	64 (6.52)	233 (25.5)
**38 months**	1211	Pearson correlation (*p*-value)			
Missing teeth	<5 *	0.013 (0.7)	0.236 (<0.001)	−0.016 (0.6)	0.103 (0.003)
Filled teeth	5 (0.41)	−0.185 (0.6)	−0.009 (0.8)	0.121 (0.0002)	0.059 (0.08)
**54 months**	1154				
Missing teeth	38 (3.29)	0.042 (0.2)	0.598 (<0.001)	0.164 (<0.001)	0.284 (<0.001)
Filled teeth	60 (5.20)	0.239 (<0.001)	0.150 (<0.001)	0.550 (<0.001)	0.359 (<0.001)
**5.5 years**	1073				
Missing teeth	37 (3.45)	0.104 (0.002)	0.493 (<0.001)	0.034 (0.3)	0.297 (<0.001)
Filled teeth	102 (9.51)	0.393 (<0.001)	0.102 (0.004)	0.464 (<0.001)	0.457 (<0.001)

* corresponding percentages not reported because of the small cell size. Dmft-decayed missing and filled teeth index.

**Table 5:** Measures of correlation between subjective reports of missing and filled teeth with examiner determined missing and filled teeth at different time points, according to selected factors: Avon Longitudinal Study of Parents and Children.

Variable	31 vs. 38 Months		43 vs. 54 Months				5 vs. 5.5 Years			
	Missing Teeth		Missing Teeth		Filled Teeth		Missing Teeth		Filled Teeth	
	Correlation (95% CI)	*p*-Value	Correlation (95% CI)	*p*-Value	Correlation (95% CI)	*p*-Value	Correlation (95% CI)	*p*-Value	Correlation (95% CI)	*p*-Value
**Age at Delivery**		<0.0001		<0.0001		<0.0001		0.0003		0.02
<30 years	0.35 (0.28, 0.41)		0.17 (0.09,0.25)		0.10 (0.02, 0.17)		0.39 (0.32, 0.46)		0.52 (0.46, 0.58)	
≥30 years	−0.003 (−0.003, −0.1)		0.46 (0.39, 0.52)		0.51 (0.44, 0.57)		0.56 (0.50, 0.62)		0.41 (0.33, 0.48)	
**Education**		<0.0001		<0.0001		<0.0001		0.14		0.9
≤O level	−0.005 (−0.08, 0.07)		0.20 (0.13, 0.27)		0.20 (0.13, 0.27)		0.53 (0.47, 0.59)		0.46 (0.39, 0.52)	
≥A level	0.56 (0.49, 0.62)		0.56 (0.49, 0.62)		0.52 (0.45, 0.58)		0.46 (0.38, 0.53)		0.47 (0.39, 0.54)	
**Child Sex**		<0.0001		0.004		0.00003		0.001		0.8
Male	−0.01 (−0.09, 0.25)		0.34 (0.27, 0.41)		0.22 (0.14, 0.29)		0.54 (0.48, 0.60)		0.46 (0.39, 0.52)	
female	0.71 (0.66, 0.75)		0.18 (0.10, 0.26)		0.44 (0.37, 0.51)		0.38 (0.30, 0.45)		0.47 (0.40, 0.54)	
**Child Ever Visited a Dentist**				<0.0001		0.9		0.0002		0.55
Yes	n.e.		0.27 (0.21, 0.33)		0.33 (0.27, 0.39)		0.47 (0.42, 0.52)		0.47 (0.42, 0.52)	
No	n.e.		0.70 (0.62, 0.77)		0.34 (0.21, 0.46)		0.70 (0.62, 0.77)		0.43 (0.30, 0.54)	

Heterogeneity *p*-value, n.e. not estimable. CI-Confidence Interval.

## Declarations

Ethics approval and consent to participate: The study was approved by the Virginia Commonwealth University Institutional Review Board as exempt (IRB #: HM20011742) on 26 October 2017. Availability of data and materials: The datasets generated and/or analyzed during the current study are not publicly available but can be requested in writing from the AVON study http://www.bristol.ac.uk/alspac/researchers/access/. The study website contains details of all the data that are available through a fully searchable data dictionary and a variable search tool at http://www.bristol.ac.uk/alspac/researchers/our-data/.

## Abbreviations

ALSPAC	Avon Longitudinal Study of Parents and Children
CIF	Children in Focus
DMFT	Decayed, Missing, Filled index
